# Does Metaphyseal Cement Augmentation in Fracture Management Influence the Adjacent Subchondral Bone and Joint Cartilage? An In Vivo Study in Sheep Stifle Joints

**DOI:** 10.1097/MD.0000000000000414

**Published:** 2015-01-26

**Authors:** Michael Goetzen, Ladina Hofmann-Fliri, Daniel Arens, Stephan Zeiter, Vincent Stadelmann, Dirk Nehrbass, R. Geoff Richards, Michael Blauth

**Affiliations:** From the AO Research Institute Davos (MG, L-HF, DA, SZ, VS, DN, GR), Davos, Switzerland; and Department of Trauma Surgery (MG, MB), Medical University of Innsbruck, Innsbruck, Austria.

## Abstract

Augmentation of implants with polymethylmethacrylate (PMMA) bone cement in osteoporotic fractures is a promising approach to increase implant purchase. Side effects of PMMA for the metaphyseal bone, particularly for the adjacent subchondral bone plate and joint cartilage, have not yet been studied. The following experimental study investigates whether subchondral PMMA injection compromises the homeostasis of the subchondral bone and/or the joint cartilage.

Ten mature sheep were used to simulate subchondral PMMA injection. Follow-ups of 2 (4 animals) and 4 (6 animals) months were chosen to investigate possible cartilage damage and subchondral plate alterations in the knee. Evaluation was completed by means of high-resolution peripheral quantitative computed tomography (HRpQCT) imaging, histopathological osteoarthritis scoring, and determination of glycosaminoglycan content in the joint cartilage. Results were compared with the untreated contralateral knee and statistically analyzed using nonparametric tests.

Evaluation of the histological osteoarthritis score revealed no obvious cartilage damage for the treated knee; median histological score after 2 months 0 (range 4), after 4 months 1 (range 5). There was no significant difference when compared with the untreated control site after 2 and 4 months (*P* = 0.23 and 0.76, respectively). HRpQCT imaging showed no damage to the metaphyseal trabeculae. Glycosaminoglycan measurements of the treated joint cartilage after 4 months revealed no significant difference compared with the untreated cartilage (*P* = 0.24).

The findings of this study support initial clinical observation that PMMA implant augmentation of metaphyseal fractures appears to be a safe procedure for fixation without harming the subchondral bone plate and adjacent joint cartilage.

## INTRODUCTION

Implant augmentation with polymethylmethacrylate (PMMA) has recently gained popularity as a promising approach to reduce cutout and provide better implant anchorage in osteoporotic fracture management. Most studies dealing with implant augmentation are based on biomechanical tests.^1–5^ Developments of the augmentation technique led to a standardized procedure, so clinical approval was assigned in 2011 for the proximal femoral nail antirotation (PFNA, Synthes GmbH, Oberdorf, Switzerland).^6^ Kammerlander et al^7^ have recently demonstrated first promising clinical results of augmentation of the proximal femur. Nevertheless, potential PMMA cement-related side effects have only been investigated to a certain extent. Pitfalls such as heat necrosis of bone tissue during polymerization or pressure generation in the medullary cavity during injection proved to be unfounded in ex vivo studies.^8–10^ To date, it is unclear and not thoroughly investigated if cement penetrating the subchondral bone area is inducing any damage to the closely adjacent articular hyaline cartilage.

**Figure 1:**
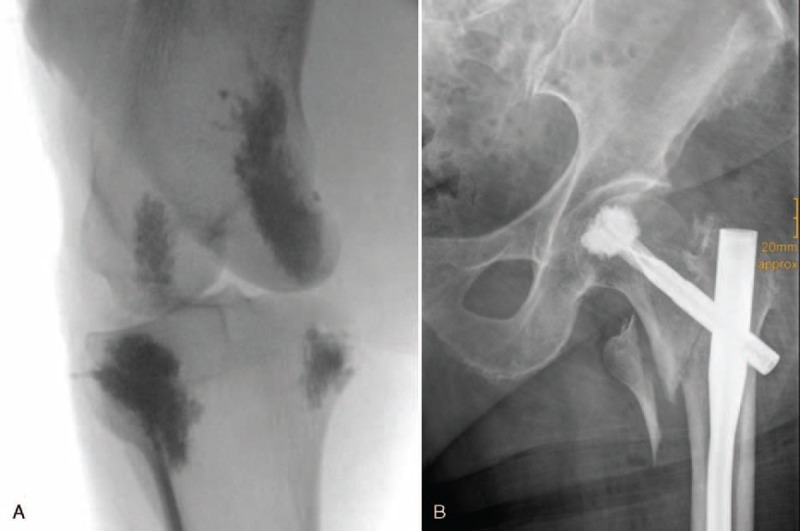
(A) Placement of 2 large (approximately 2 mL) and two small (approximately 0.5 mL) depots of bone cement in the sheep knee joint. (B) Clinical example of subchondral cement distribution after PFNA blade augmentation. PFNA = proximal femoral nail antirotation.

Sermon et al^2^ investigated cement distribution in cadaveric human femoral heads after PMMA injection through the PFNA blade and could not find any regular pattern. In fact, cement was often seen very close to the subchondral plate. A clinical example is shown in Figure 1B.

A hard subchondral cement layer could lead to a different force distribution along the cartilage and the subchondral bone. Newly increased shear forces at the cement bone interface could lead to microcracks in the subchondral bone and fissures in the cartilage.^11,12^

Furthermore, the subchondral bone is a permeable layer that allows the transport of nutrients from the bone marrow to the overlying cartilage. Several authors have shown that this nutritional pathway can majorly affect the sensitive cartilage homeostasis.^13–18^ An interruption or disruption of this pathway, due to subchondral augmentation, could lead to critical shortage of the cartilage nutrition. Due to these concerns, the overall response to implant augmentation in clinics is still cautious, since possible joint cartilage violation through subchondral PMMA injection has not been ruled out yet.

Several in vivo studies using different animal models investigated the effect on articular cartilage of subchondral bone replacement with autologous bone, PMMA, or calcium phosphate cements.^19–21^ However, the applied methodology (type, amount of cement, and location) differs between studies and the drawn conclusions vary widely.

Conversely, hyaline cartilage reactions have been assessed in clinical studies investigating PMMA filling of voids after giant cell tumors around the knee. No evidence was found that presence of cement close to the knee joint was associated with the development of degenerative osteoarthritis.^22,23^ However, the radiographic quantification of cartilage degeneration is not very accurate, especially in early stages and in localized lesions.^24^ Furthermore, due to the absence of nerves in the cartilage and the replacement of the complete subchondral bone area with PMMA, the situation is rather different from an intact knee joint with some augmentation material beneath.

To date, all the above-mentioned studies analyzing possible negative effects of biomaterials on adjacent cartilage have examined void filling procedures after defect creation, but did not investigate exclusive augmentation of metaphyseal cancellous bone areas in vivo.

To assure safe implant augmentation, the objective of this study was to investigate the effect of PMMA augmentation on the subchondral plate and the overlying joint cartilage in vivo.

## METHODS

### Study Design

The animal model used in this study is the ovine stifle joint and is similar to the model described by Klein et al.^25^ Each animal received 4 cement injections with varying volumes and distances to the cartilage into the right distal femur and proximal tibia. The contralateral left knee served as untreated control site. Regions of interest (ROIs) were evaluated separately for lateral (ROI 1) and medial joint area (ROI 2). The ROIs were defined where the cement distribution occurred most subchondral at the center of gravity of the cement cloud within the weight-bearing joint surface. Evaluation was based on a macroscopic and histological evaluation using a modified osteoarthritis score, biochemical determination of glycosaminoglycan (GAG) content, and high-resolution quantitative computed tomography (CT) imaging of the cement cloud and the surrounding trabecular bone meshwork. This study was performed according to the Swiss animal welfare regulations and approved by the ethical committee of the canton Graubünden, Switzerland (No. 2012_29).

### Animals

Ten skeletally mature female Swiss Alpine sheep, aged 2 to 4 years, weighing 69 ± 10 kg were selected. The animals were clinically and radiologically examined by a veterinarian prior to the start of the study to rule out existing orthopedic disorders. Sheep were acclimatized to postsurgical conditions at least 2 weeks prior to surgical intervention. The animals were fed twice a day with silage, hay, and straw. They always had free access to drinking water.

### Surgery

The entire surgical procedure was performed under aseptic conditions while the animals were placed under general anesthesia.

The sheep were sedated with 0.05 mg/kg detomidine (Domosedan, Pfizer AG, Zürich, Switzerland) intramuscularly while they were still in the stable. Induction was done using 0.2 mg/kg midazolam (Dormicum, Hoffmann-La Roche, Basel, Switzerland) and 6 mg/kg ketamine (Ketasol-100, Dr. E. Graeub AG, Berne, Switzerland) intravenously. Anesthesia was maintained using approximately 1.5% isoflurane (Isoflurane Baxter, Baxter AG, Volketswil, Switzerland) in oxygen (oxygen flow rate between 0.6 and 1 L/min). Preemptive analgesia was conducted using 1.4 mg/kg carprofen (Rimadyl Rind, Zoetis, Zürich, Switzerland) intravenously and epidural anesthesia with 1 mL buprenorphine (0.3 mg/mL Temgesic, Reckitt Benckiser AG, Wallisellen, Switzerland) mixed with 5 mL lidocaine 2% (Lidocain 2%, Streuli Pharma AG, Uznach, Switzerland). Each animal received as perioperative antibiotics 2.2 mg/kg ceftiofur intravenously (Excenel, Zoetis) 1 hour before the first surgical incision.

Using a standardized aiming device (combined aiming device: 130.30, DePuy Synthes Vet, West Chester, PA, USA), 2 drill holes with a 2.5 mm drill bit were placed at alternating distances of approximately 5 or 10 mm to the joint surface beneath the medial and lateral tibial plateau. The same procedure was performed in the distal femur. Cannulas with 2.5 mm front opening (Unimed, Lausanne, Switzerland) were inserted into the 4 drill holes and 4 depots of Traumacem V+ (REF 07.702.040S, DePuy Synthes, Oberdorf, Switzerland) were injected as follows: 0.5 mL (=“small” cement volume) in approximately 5 or 10 mm distance to the joint line, respectively, and 2 mL (=“large” cement volume) in approximately 5 or 10 mm distance to the joint line, respectively. The radiographs in Figure 1 show the 4 cement depots after injection and a clinical example of subchondral cement augmentation.

Postoperatively, sheep were kept in individual pens for 1 day, until they were group housed. Sheep were allowed to fully weight bear immediately after surgery. To alleviate acute postoperative pain, the animals were given 1.4 mg/kg carprofen (Rimadyl) every third day for 5 days, buprenorphine (Temgesic) 0.01 mg/kg 3 times a day for 24 hours, and fentanyl Patches (Durogesic Matrix) 2 μg/kg/h for 72 hours. Sutures were removed after 14 days.

### Euthanasia and Sample Harvest

The animals were euthanized by means of intravenous administration of pentobarbital (300 mg/mL; Esconarkon, Ad. Us.Vet.). Four animals were euthanized after a follow-up of 2 months and 6 animals after 4 months. Tibiae and femora of both stifle joints of each animal were harvested immediately after euthanasia. Evaluation of the cartilage was first performed macroscopically using the modified International Cartilage Repair Society (ICRS) grading system for chondral injuries at the time point of the necropsy (Table 1).^26^ Scoring was performed within 30 minutes after harvesting on the intact bone prior to scanning and histological processing.

**Table 1:**
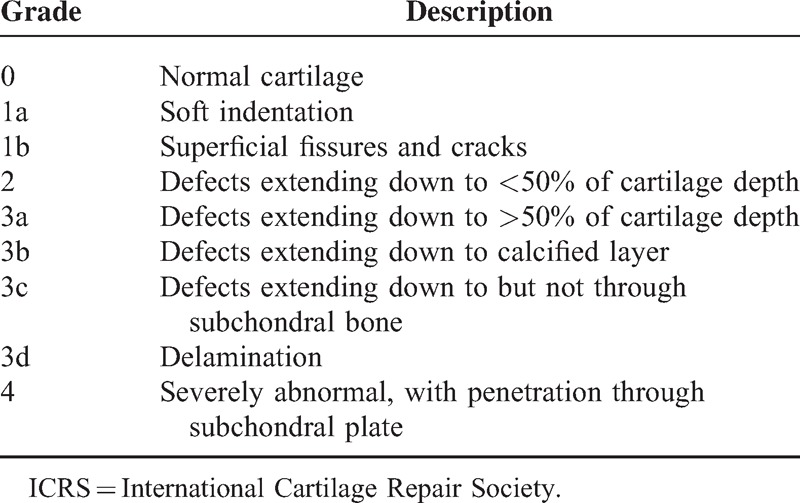
Macroscopic Scoring According to the ICRS Grading System for Chondral Injuries^26^

### High-Resolution Peripheral Quantitative Computed Tomography (HRpQCT)

The fresh, unfixed proximal tibiae and distal femora were scanned using a HRpQCT (XtremeCT, SCANCO Medical AG, Brüttisellen, Switzerland) at an isotropic resolution of 82 μm to evaluate actual cement distribution in situ. 3D evaluations allowed volumetric calculation of the injected cement in situ. Trabecular structure in the subchondral bone was assessed qualitatively.

### Histology

The harvested bones were cut along the ROIs in the frontal plane to achieve best view of the cement–subchondral bone and subchondral bone–cartilage interface. Samples were fixed in 70% ethanol, dehydrated, and embedded in LR White and 2 slices per sample, with a distance of 1000 μm, were cut on a saw microtome and stained with Giemsa–eosin as well as Safranin O.

An osteoarthritis grading and staging system, related to Mankin histological score,^27–29^ was used to assess alterations compared with the control site (Table 2). A certified veterinarian pathologist and an orthopedic surgeon performed scoring using a light microscope. The osteoarthritis grading system focused on structural alterations in the hyaline joint cartilage dividing into the noncalcified and calcified layers. Safranin O staining was used to assess proteoglycan content in the cartilage. Diminishing staining intensity indicates loss of proteoglycan as seen in osteoarthritic changes in the cartilage. Tidemark integrity was investigated at the subchondral bone and calcified cartilage interface.^27,30^ Above each ROI, average subchondral plate thickness was measured and for the treated samples additionally the average distance between cement cloud and cartilage was calculated.

**Table 2:**
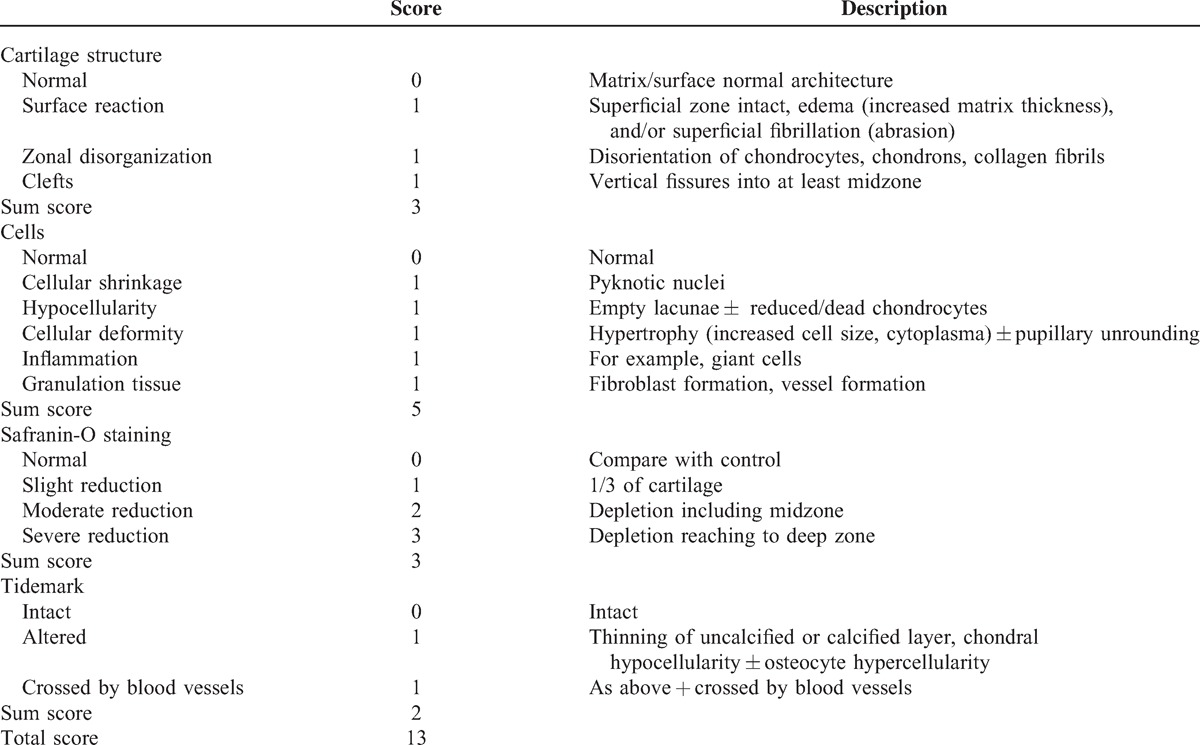
Modified Mankin Score for Hyaline Cartilage

### Biochemistry

Biochemical evaluation was performed only after 4 months. Tissue samples were harvested with the help of a biopsy punch (0.4 mm diameter) above the ROIs. Samples were digested in proteinase K and measurement of sulfated GAG using 1.9-dimethylmethylene blue assay (Sigma, Buchs, Switzerland) was performed. Samples were normalized to DNA content (PicoGreen assay, Invitrogen, Zug, Switzerland).

### Statistics

Statistics was performed using SPSS (SPSS 22, IBM Corporation, New York, NY). After assessing data distribution (Shapiro–Wilk), paired nonparametric test statistics (Wilcoxon signed-rank) were performed to identify differences between treated and untreated control samples regarding histological score, subchondral plate thickness, and GAG content ratio. Spearman correlation coefficient *R*^2^ was used to assess correlations between histological score and cement volume and distance. *P* values of <0.05 were accepted as significant.

## RESULTS

In the lateral tibial plateau of 1 sheep of the 2-month follow-up group it was not possible to place the cement at the weight-bearing area. Therefore, this ROI was excluded from evaluation, leaving 15 ROIs that could be evaluated for the 2-month follow-up group. Macroscopic evaluation using ICRS grading system revealed for 1 sheep of the 4-month follow-up group a grade of 3a at the medial tibial plateau and medial femur condyle. Also a grade of 4 was given for the lateral femur condyle. Those findings relied on an intra-articular cement extravasation as suspected during surgery and documented in the surgery report. Those ROIs were excluded from further evaluation, leaving 21 ROIs that could be evaluated for the 4-month follow-up group. Two more ROIs from 4-month sheep were given a grade of 2 and 3a, respectively, whereas the defects were suspected to be caused iatrogenically during bone harvest. These ROIs were kept for further evaluation since microscopic evaluation would be unaffected by such defects. For all the remaining ROIs and animals, evaluation revealed no macroscopic changes to the hyaline cartilage (grade 0).

### HRpQCT Evaluation

**Figure 2:**
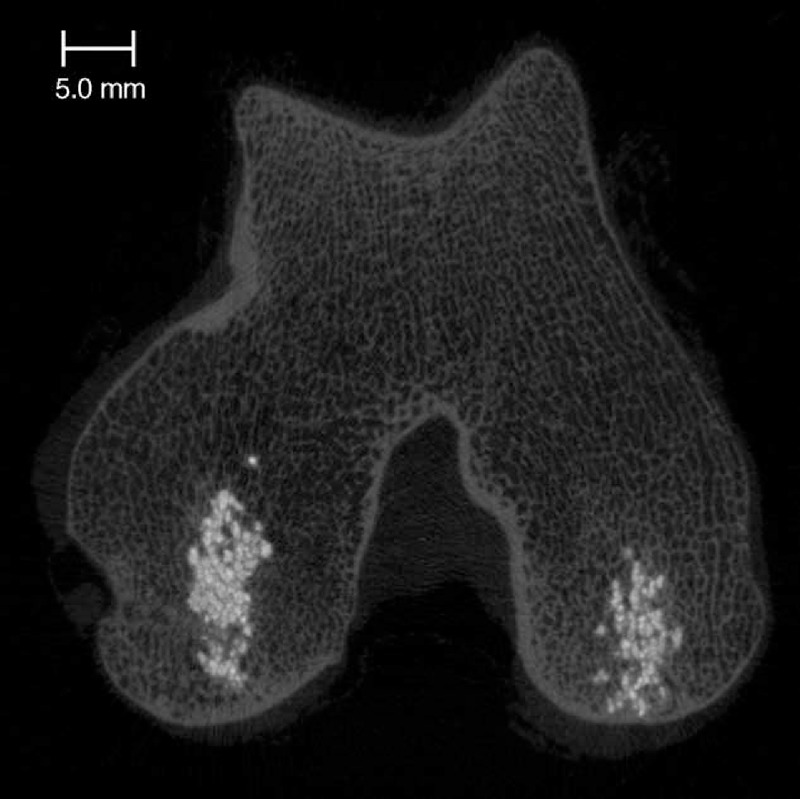
XtremeCT section showing the cement distributing around the trabeculae in the femoral condyles.

HRpQCT scans did not reveal breakage of the trabeculae. The cement distributed between the trabeculae, preserving their fine meshwork (Figure 2). Measurements of the cement volume in situ revealed an average of 2.01 ± 0.24 mL for the large cement volume and 0.4 ± 0.11 mL for the small cement volume in the 2-month animals. In the 4-month animals, the large cement volume was 2.14 ± 0.34 mL and the small cement volume was 0.51 ± 0.15 mL. No relation was found between cement volume and histological score, neither in the 2-month samples (*P* = 0.30, *R*^2^ = 0.08) nor in the 4-month samples (*P* = 0.10, *R*^2^ = 0.14).

### Histology

**Figure 3:**
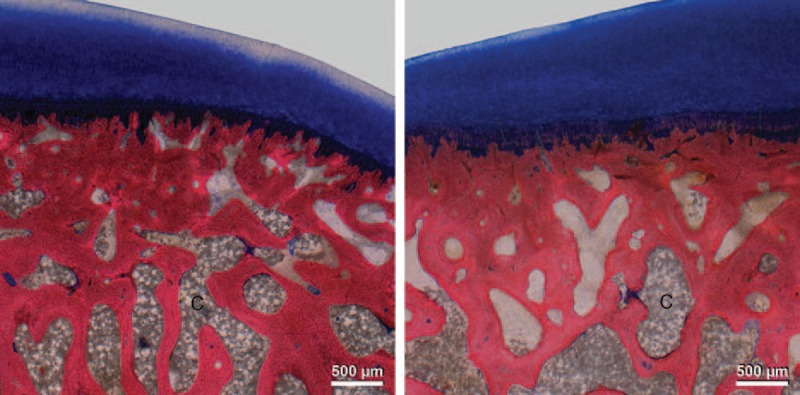
Giemsa–eosin-stained sections showing subchondrally injected bone cement (C) and the overlying cartilage: no obvious cartilage alterations after 2-month follow-up (left) and after 4-month follow-up (right) with a large amount of cement very close to the cartilage.

**Figure 4:**
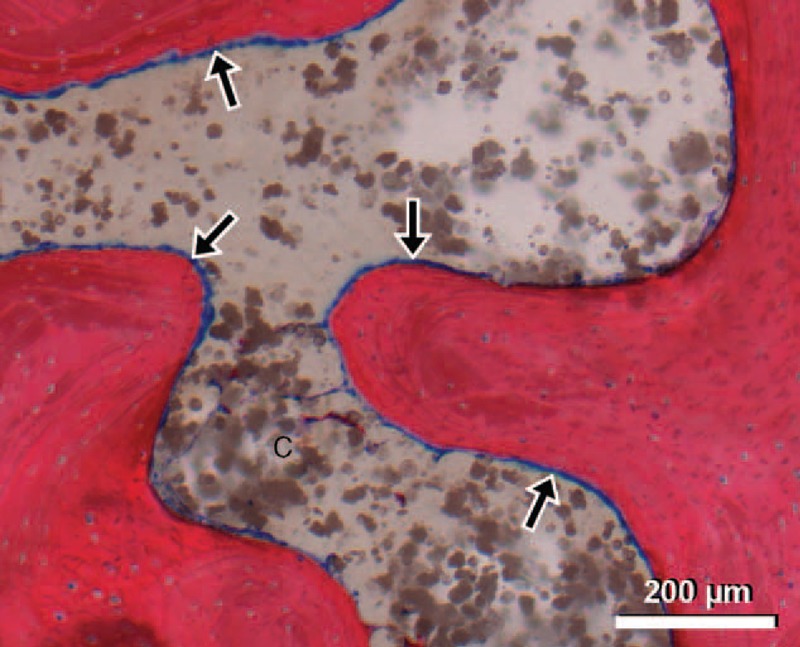
Giemsa–eosin-stained section showing osteocyte reaction to bone cement (C). Arrows indicate high presence of osteoblasts at the cement–bone interface (2-month follow-up).

Table 3 summarizes the results of the histological evaluation. No statistical significant difference was found in the modified Mankin score between the treated and untreated sites after 2 months (*P* = 0.23) and after 4 months (*P* = 0.76). Figure 3 shows the histological sections of “worst-case” samples (large amount of cement very close to the cartilage) from both time points. Measurement of the subchondral plate thickness showed no significant difference between the sites after 2 months (*P* = 0.57) and 4 months (*P* = 0.59). A correlation between cement distance to cartilage and histological score was not observed in the 2-month samples (*P* = 0.13, *R*^2^ = 0.16) and also the 4-month samples showed no such relation (*P* = 0.44, *R*^2^ = 0.03). Inside the cement cloud, an osseous reaction, characterized by a slightly higher trabecular lining density of osteoblasts, was observed after 2 and 4 months (Figure 4). Also in the center of the large cement clouds (2 mL), a relatively high number of osteoblasts were observed.

**Table 3:**
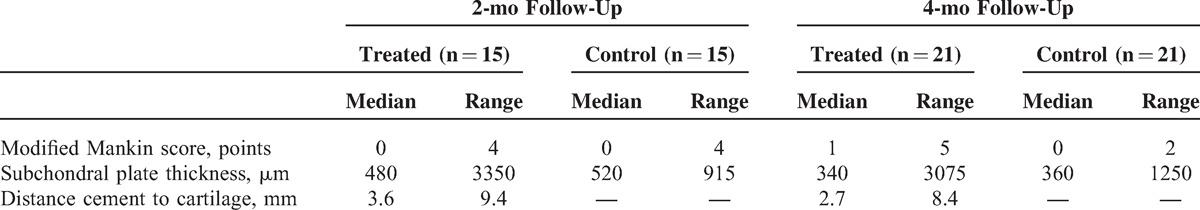
Overview of Histology Results

### Biochemistry

Median GAG content ratio in the cartilage above the cemented area after a follow-up of 4 months was 168 (range 616) compared with 100 (range 730) for the untreated site with no difference between the 2 sites (*P* = 0.24).

## DISCUSSION

Bone cement augmentation appears to be a promising solution to increase implant purchase in osteoporotic fractures. Nevertheless, the effect of subchondral PMMA-based bone cement on the nearby cartilage has not yet been investigated. In this in vivo sheep study, possible changes in the subchondral bone and the adjacent joint cartilage were assessed by means of HRpQCT imaging, macroscopic and microscopic scoring, and biochemical evaluation of the subchondral bone plate and cartilage.

The results of this study revealed no critical cartilage damage as far as 4 months after subchondral augmentation of up to 2 mL PMMA in sheep stifle joints.

In a study using 2 techniques for bone marrow stimulation of cartilage repair Chen et al^31^ demonstrated that due to compaction of bone during microfracture the osteocytes are subjected to direct mechanical damage and shear that in consequence induces cell death. Therefore, one concern was that compaction of subchondral bone due to cement augmentation can induce cartilage damage. In this study neither HRpQCT evaluation nor light microscopic evaluation did confirm obvious breakage of the trabecular structure in the evaluated subchondral bone; in fact, cement distributed into the intertrabecular space. The high force that a surgeon sometimes needs during cement injection does not seem to reflect the pressure with which the cement is pressed through the trabecular bone. This is in accordance with the conclusion by Blankstein et al,^8^ who measured the pressure in human femoral heads during injection of bone cement in vitro. The different quality of sheep bone and osteoporotic human bone should be taken into account; however, Braunstein et al observed similar behavior with means of contact radiography, computed tomography, and scanning electron microscopy in augmented osteoporotic vertebral bodies.^32^ Furthermore, this was confirmed by a qualitative analysis of HRpQCT scans of cement-augmented osteoporotic human cadaveric bones from previous augmentation studies.^2,3,10^

Microscopic evaluation revealed no significant differences between the treated and untreated limbs when comparing the osteoarthritis score after 2 and 4 months. The measured cartilage changes seem to be physiological according to Armstrong et al.^33^ They scored normal, untreated sheep stifle joints using the Mankin score to show topographical alterations and described slight roughening and fissuring as physiological. Vandeweerd et al^34^ also advise to take into account prevalent subclinical cartilage defects at the baseline in studies using ovine models.

Another reason why subchondral PMMA augmentation was questioned was a suspected disruption of the nutrition pathway from the intramedullary cavity to the cartilage. While cartilage and subchondral bone remained unaffected by the bone cement, the trabecular bone within the cement cloud, however, showed a mild reaction. Interestingly, this osteoblast hypercellularity was even observed in the center of the large cement clouds. This suggests that cell viability and blood supply of the adjacent bone and cartilage might not be compromised by the presence of PMMA and could explain the different results from other studies where a defect was created before the cement was placed near the subchondral bone.^17,20^

It is believed that higher loads during the development of osteoarthritis, due to cartilage thinning, increase bone turnover in the subchondral bone whereby cytokines are released through the calcified cartilage, which in turn leads to the progression of osteoarthritis.^13,35,36^ Cementing of the trabecular meshwork reduces its elasticity, resulting in higher loads to the bone. Similar pathophysiology could explain the increased cell number within the cement cloud. The used PMMA, Traumacem V+ (DePuy Synthes, Oberdorf, Switzerland), contains 10% hydroxyapatite. Osseous cell accumulation could therefore also be interpreted as bony reaction to the applied exogenic hydroxyapatite. The exact reason for these changes within the cement cloud, to what extent they continue and to what they lead, needs to be investigated in a separate study with a longer follow-up.

The subchondral plate and the underlying fine trabecular network of the metaphysis keep the balance to provide stiffness and to buffer axial peak forces transduced through the hyaline cartilage.^37^ Another concern of subchondral augmentation was that stiff bone cement could affect this system. Cartilage damage might occur similar to the processes in osteoarthritis, where stiffness of the subchondral bone is increased through sclerosis.^38–40^ Additional shear forces are generated when a discontinuity in stiffness, such as subchondral changes, occurs.^41^ Similar to that, one might expect a possible tear of the cartilage or crack in the subchondral bone at the interface between the cement cloud and the normal bone. Microscopic evaluation revealed none of these findings. Density and thickness of the subchondral bone show a large variation across the joint surface.^33^ This is due to the fact that stress to cancellous bone causes buckling of the trabeculae and induces remodeling to strengthen the loaded area.^39,42^ Microscopic and HRpQCT analysis did not show buckling of the trabeculae due to the cement. After 2 and 4 months, subchondral plate thickness showed no significant difference between the treated and untreated sites.

Early degradation of cartilage in osteoarthritis is described as loss of GAG content.^41,43^ After 4 months, the biochemical evaluation of the GAG content ratio in the cartilage above the cement was not different from the untreated control site. This indicates that also early changes in the cartilage that could not have been detected by histological means can be ruled out after a follow-up of 4 months.

The ovine stifle joint has proved to be a good model to investigate and simulate osteoarthritis, regeneration of partial- and full-thickness osteochondral defects, and to test orthopedic implants. The sheep stifle joint model was used for this study for the following 3 reasons. A large animal model with an average weight of the sheep of 69.2 kg (standard deviation 10.38) better simulates loads in the human knee joint than small animal models. Dimensions of the sheep knee are one-third of the human knee,^44^ thus allowing the injection of up to 3 mL cement per bone in cadaver trials, as used for the PFNA augmentation. In this study, a maximum of 2 mL was injected; however, considering the relative amount of cement with respect to the joint size this is a clinically relevant volume. The knee was chosen instead of the hip because of the easy and less invasive surgical approach.^45^ Because it is very difficult to measure an exact percentage filling of subchondral bone, especially in clinics, we decided to focus on the distance of the cement cloud to the cartilage. This distance was small enough to assure that several samples had cement penetrating the subchondral bone. We believe that potential regulatory disturbances could have been detected with this model.

One drawback of the study is the short follow-up. Malinin and Ouellette^17^ investigated the integration of PMMA-coated osteochondral autografts in the subchondral bone and compared it with a control autograft placed into vascularized, viable cancellous bone of baboons. After a follow-up of 5 and 12 months, they found only little effects on the cartilage, whereas after 3 years, degenerative changes comparable with osteoarthritis were observed. It needs to be considered that the autograft was totally disconnected to the underlying subchondral bone. In our study, we showed that PMMA injected subchondrally without previous void creation retains the subchondral bone structure and the viability of the trabecular bone within the cement seems to be preserved. Another study also detected characteristic macroscopic joint changes in an osteoarthritis model by lateral meniscectomy already after 3 months.^46^

In conclusion, the findings of this study showed that the injection of PMMA-based bone cement close to the joint line does not seem to damage the adjacent subchondral bone or cartilage after an observation period of up to 4 months in sheep. Hypercellularity within the cement cloud was even observed without any effect for the adjacent joint cartilage. First clinical long-term results of PFNA blade augmentation with PMMA have recently been published by Kammerlander et al.^7^ They found no cartilage or bone necrosis after 15 months when evaluating clinical scores and computed tomography scans. PFNA augmentation seems to be a clinically safe procedure for the treatment of osteoporotic pertrochanteric fractures without chances of harming the adjacent joint cartilage. New cement augmentation models for various osteoporotic fracture types and locations are currently under investigation. This study suggests that short-term to midterm negative effects of the PMMA for the joint cartilage can be ruled out.
